# Pancreatic gangliocytic paraganglioma harboring lymph node metastasis: a case report and literature review

**DOI:** 10.1186/s13000-017-0648-x

**Published:** 2017-08-02

**Authors:** Keisuke Nonaka, Yoko Matsuda, Akira Okaniwa, Atsuko Kasajima, Hironobu Sasano, Tomio Arai

**Affiliations:** 1 Department of Pathology, Tokyo Metropolitan Geriatric Hospital, 35-2 Sakae-cho, Itabashi-ku, Tokyo, 173-0015 Japan; 2 Department of Pathology, Tohoku University School of Medicine, 2-1 Seiryou-machi, Aoba-ku, Sendai-shi, 980-8575 Japan; 3 Department of Surgery, Kimitsu Chuo Hospital, 1010 Sakurai, Kisarazu, Chiba, 292-8535 Japan

**Keywords:** Pancreas, Gangliocytic paraganglioma, Neuroendocrine tumor grade 1, Adrenocortical adenoma, Case report

## Abstract

**Background:**

Gangliocytic paraganglioma (GP) is a rare neuroendocrine neoplasm, which occurs mostly in the periampullary portion of the duodenum; the majority of the reported cases of duodenal GP has been of benign nature with a low incidence of regional lymph node metastasis. GP arising from the pancreas is extremely rare. To date, only three cases have been reported and its clinical characteristics are largely unknown.

**Case presentation:**

A nodule located in the pancreatic head was incidentally detected in an asymptomatic 68-year-old woman. Computed tomography revealed 18-, 8-, and 12-mm masses in the pancreatic head, the pancreatic tail, and the left adrenal gland, respectively. Subsequent genetic examination revealed an absence of mutations in the *MEN1* and *VHL* genes. Macroscopically, the tumor located in the pancreatic head was 22 mm in size and displayed an ill-circumscribed margin along with yellowish-white color. Microscopically, it was composed of three cell components: epithelioid cells, ganglion-like cells, and spindle cells, which led to the diagnosis of GP. The tumor was accompanied by a peripancreatic lymph node metastasis. The tumor in the pancreatic tail was histologically classified as a neuroendocrine tumor (NET) G1 (grade 1, WHO 2010), whereas the tumor in the left adrenal gland was identified as an adrenocortical adenoma. The patient was disease-free at the 12-month follow-up examination.

**Conclusions:**

Pancreatic GP is associated with a higher incidence of metastasis and larger tumor size than duodenal GPs, suggesting that the primary organ of GP is an important prognostic factor.

## Background

Since first report in 1957 [1], gangliocytic paragangliomas (GPs) have been found to occur mostly in the periampullary portion of the duodenum [2], although some have been observed in the jejunum [3], pylorus [4], esophagus [5], nasopharynx [6], ovary [7], and lung [8]. The pancreas is also an extremely rare site of GP; only three cases have been reported to date, making the clinical characteristics of pancreatic GP unclear [9–11]. GPs are typically associated with an extremely good prognosis, but patients harboring lymph node metastases have occasionally been reported [12]. Radical surgery with lymph node dissection is therefore the mainstay of curative treatment [13]. GPs consist of three characteristic tumor components: epithelioid, spindle, and ganglion-like cells [14]. Histopathologically, it can be challenging to distinguish pancreatic GPs from other pancreatic neoplasms with neuroendocrine differentiation, such as paragangliomas and neuroendocrine tumors, because of their low incidence, rare location, and similar morphological and immunohistochemical characteristics [15, 16]. Their clinical presentation and treatment strategy, however, appear to differ [11, 17, 18], making precise differentiation crucial for pathological diagnosis.

This report describes a patient with pancreatic GP harboring lymph node metastasis. The patient was also found to have a pancreatic neuroendocrine tumor (NET) G1 (grade 1, WHO 2010) and an adrenocortical adenoma. Herein, the clinical characteristics of pancreatic GP are reviewed, as well as the differential diagnosis of these tumors from other endocrine tumors.

## Case presentation

### Clinical history

**Fig. 1:**
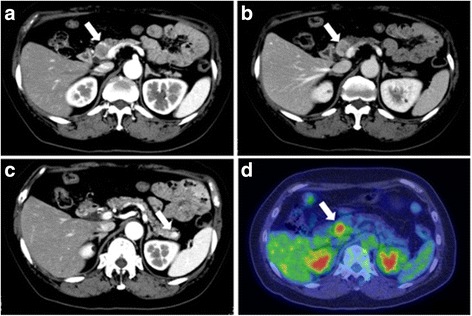
Radiological images of masses in the pancreas and the left adrenal gland. **a**-**c** Contrast-enhanced computed tomography of the pancreas. **a** Arterial phase shows a mass with peripheral enhancement (*arrow*) in the pancreatic head. **b** Portal phase demonstrates a well-defined mass with weak and heterogeneous enhancement (*arrow*) in the pancreatic head. **c** In the pancreatic tail, arterial phase reveals a rounded and well-delineated mass with marked enhancement (*arrow*). **d** Fluorine-18 fluorodeoxyglucose positron emission tomography imaging shows a positive mass (*arrow*) in the pancreatic head

An asymptomatic, 68-year-old Japanese woman with no significant past medical history was found incidentally to have a low-echoic nodule in the pancreatic head via abdominal ultrasonography. No endocrine tumors or diseases were detected in any of her family members. Her father died of stomach cancer. She neither smoked nor drank alcohol. Her blood pressure (127/69 mmHg) was well controlled via antihypertensive drugs. She was slightly obese (body mass index = 25.8 kg/m^2^). Physical examinations did not show any abnormalities. Upper gastrointestinal endoscopy did not detect any lesions in the stomach or duodenum. Endoscopic ultrasonography revealed a 21.6 mm diameter, low-echoic nodule in the pancreatic head. No dilation of the biliary or pancreatic duct was detected. On contrast-enhanced computed tomography (CT) scans, the above-described lesion was peripherally enhanced during the arterial phase (Fig. 1a) and was weakly and heterogeneously enhanced during the portal phase (Fig. 1b). In addition, CT scans identified an 8-mm mass in the pancreatic tail (Fig. 1c) with a well-defined border and marked enhancement, and a 12-mm mass in the left adrenal gland with heterogeneous enhancement. On fluorine-18 fluorodeoxyglucose (^18^F–FDG) positron emission tomography (PET) imaging, the mass in the pancreatic head demonstrated a high ^18^F–FDG uptake with a maximal standardized uptake value (SUVmax) of 4.8 (Fig. 1d), which implied a potential for malignancy. The masses in the pancreatic tail and the left adrenal gland exhibited negative ^18^F–FDG uptake. All the laboratory data was within normal limits, including serum tumor markers, carcinoembryonic antigen (CEA), carbohydrate antigen 19–9 (CA 19–9), pancreatic cancer-associated antigen-2 (Dupan-2), s-pancreas-1 antigen (Span-1), pro-gastrin-releasing peptide (Pro-GRP), and neuron-specific enolase (NSE). Pancreatic endocrine hormones were also within normal limits. Needle biopsies from the pancreatic head and tail did not provide a sufficient amount of materials for a pathological diagnosis. The patient underwent pylorus-preserving pancreaticoduodenectomy with lymph node dissection, distal pancreatectomy, and left adrenalectomy.

### Materials and methods

DNA was extracted from a peripheral blood sample and sequences corresponding to exons 2–10 of the *MEN1* gene, exons 1–3 of the *VHL* gene, and exon 16 of the *RET* gene were PCR amplified. The PCR products were directly sequenced using the BigDye Terminator v1.1 Cycle Sequencing Kit (Life Technologies, CA, USA), according to the manufacturer’s instructions, and analyzed on an automated sequencer (3130xl Genetic Analyzer, Life Technologies).

### Pathological findings

#### Gangliocytic paraganglioma in the head of the pancreas

**Fig. 2:**
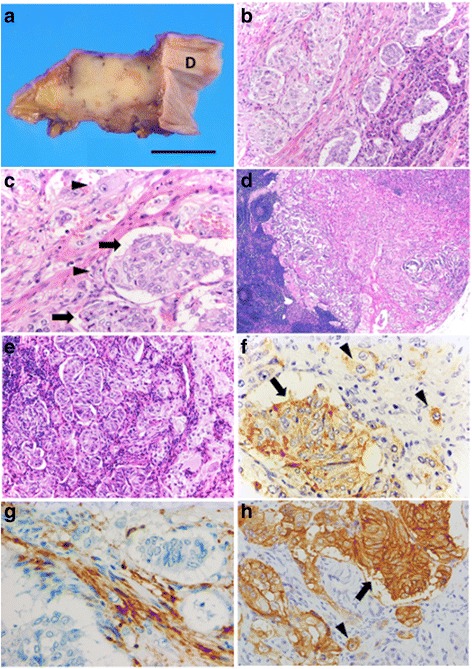
Macroscopic, histological and immunohistochemical images of gangliocytic paraganglioma. **a** The cut surface of the pancreatic head shows a poorly marginated, yellowish-white tumor, surrounded by the pancreatic tissue without involving the duodenal wall (D) (scale bar = 1 cm). **b** The tumor is seen adjacent to the pancreatic tissue (*right side*) (hematoxylin-eosin staining). **c** The tumor is composed of epithelioid cell nests (*arrow*), ganglion-like cells (*arrowhead*), and spindle cells (hematoxylin-eosin staining). **d** and **e** In the peripancreatic lymph node, the metastasizing lesion comprises all three cell types (hematoxylin-eosin staining, respectively). **f** The epithelioid (*arrow*) and ganglion-like cells (*arrowhead*) are positive for chromogranin A, whereas the spindle cells are negative. **g** Only spindle cells are immunoreactive for S-100 protein. **h** SSTR2A is expressed in the epithelioid cells (*arrow*) and in a small number of the ganglion-like cells (arrowhead) with complete membranous positivity (score 3)

Macroscopic examination revealed a poorly demarcated, solid, yellowish-white tumor, measuring 22 × 22 × 17 mm, in the head of the pancreas (Fig. 2a). Microscopically, the tumor was composed of an admixture of three cell types: epithelioid cells, ganglion-like cells, and spindle cells (Fig. 2b). The epithelioid cells, the major component of the tumor, were rather uniform in size and formed small nests (Fig. 2c), exhibiting fine granular eosinophilic cytoplasm and round-to-ovoid nuclei with stippled chromatin. This component of the tumor showed vascular, perineural, and peripancreatic adipose tissue invasion. The ganglion-like cells, the smallest component of the tumor, had vesicular nuclei, conspicuous nucleoli, and an abundance of cytoplasm (Fig. 2c). The spindle cells exhibited thin, elongated, wavy-shaped nuclei resembling Schwann cells and were arranged in intertwining fascicles. Mitosis was not conspicuous, and foci of intratumoral necrosis were not observed. The surgical margins were negative for tumor cells. Tumor tissue, composed of all the three cell types, also showed metastasis to a peripancreatic lymph node (Fig. 2d, e).

The immunohistochemical findings of the three components are summarized in Table 1. The epithelioid cells were positive for chromogranin A (Fig. 2f), synaptophysin, and pancytokeratin (AE1/AE3) but negative for catecholamine-synthesizing enzymes (tyrosine hydroxylase, dopamine β-hydroxylase, dopa decarboxylase, and phenylethanolamine *N*-methyltransferase). The ganglion-like cells were positive for synaptophysin and chromogranin A (Fig. 2f). Some of these cells were weakly positive for neurofilament, pancytokeratin, and phenylethanolamine *N*-methyltransferase. The spindle cells were positive for S-100 protein (Fig. 2g) and neurofilament. The Ki-67 labeling index (LI) of the tumor cells was 0.3%, with no apparent difference among the three cell types. The immunohistochemical features of the metastatic lesion were identical to those of the primary tumor.

**Table 1:** Immunohistochemical findings obtained from the gangliocytic paraganglioma and the neuroendocrine tumor G1

	GP in pancreatic head			NET G1 in pancreatic tail
	E	G	S	
Chromogranin A	+	+	−	+
Synaptophysin	+	+	−	+
S-100 protein	−	−	+	−
Neurofilament	−	+	+	ND
Pancytokeratin	+	+	−	ND
SSTR1	−	−	−	ND
SSTR2A	+	+	−	ND
SSTR3	−	−	−	ND
SSTR5	−	−	−	ND
Insulin	−	−	−	−
Glucagon	−	−	−	+
PP	−	−	−	−
Serotonin	−	−	−	ND
Somatostatin	+	+	−	−
Tyrosine hydroxylase	−	−	−	ND
Dopamine β-hydroxylase	−	−	−	ND
Dopa decarboxylase	−	−	−	ND
Phenylethanolamine *N*-methyltransferase	−	+	−	ND
PgR	+	+	−	+

*GP* gangliocytic paraganglioma, *NET* neuroendocrine tumor, *G1* grade 1, *E* epithelioid cells, *G* ganglion-like cells, *S* spindle cells, *SSTR* somatostatin receptor, *PP* pancreatic polypeptide, *PgR* Progesterone receptor, + positive, − negative; *ND* Not done

We also investigated the immunohistochemical expression of hormonal products and hormone receptors (Table 1). Somatostatin receptor (SSTR) 2A was expressed on the cell membranes of more than 50% of epithelioid cells (Fig. 2h), which was defined as a score of 3 [19]. SSTR2A expression was also detected in a small number of ganglion-like cells (Fig. 2h). The epithelioid cells and some ganglion-like cells were positive for progesterone receptor (PgR). All the three cell types were negative for pancreatic polypeptide (PP).

#### Neuroendocrine tumor in the tail of the pancreas

**Fig. 3:**
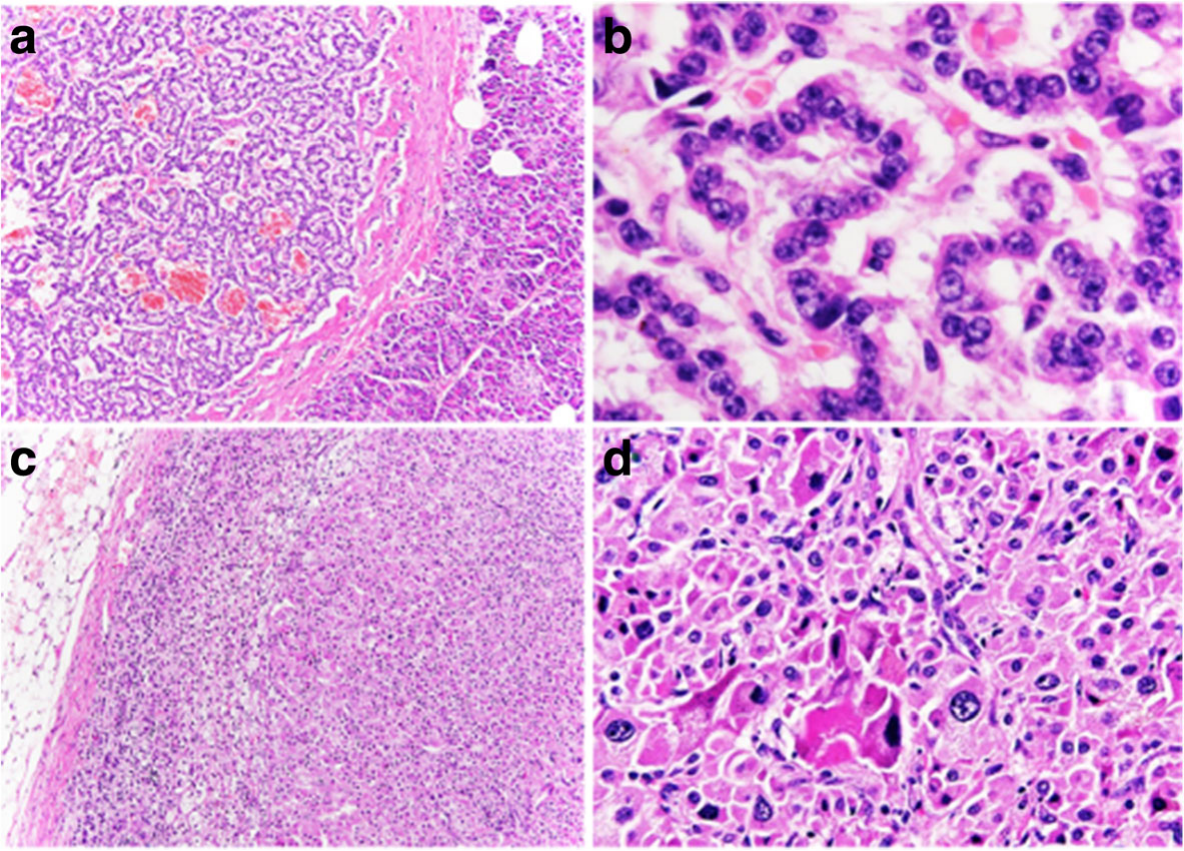
Histological images of the tumor in the pancreatic tail and left adrenal gland. **a** The pancreatic tail tumor shows trabecular and ribbon-like growth patterns with fibrovascular stroma (hematoxylin-eosin staining). **b** The pancreatic tail tumor exhibits the following cytological features; small round nuclei, a salt-and-pepper chromatin pattern, and an eosinophilic, finely granular cytoplasm (hematoxylin-eosin staining). **c** The adrenal tumor is well-circumscribed and nonencapsulated (hematoxylin-eosin staining). **d** The adrenal tumor shows lipid-poor compact cells with an eosinophilic cytoplasm (hematoxylin-eosin staining)

A well-demarcated, grayish-white tumor, 7 × 7 mm in size was found in the pancreatic tail. Microscopically, the tumor was composed of round-to-columnar cells with uniformly round nuclei, a salt-and-pepper chromatin pattern, and an eosinophilic, finely granular cytoplasm, arranged in trabecular and ribbon-like patterns and accompanied by fibrous stroma (Fig. 3a, b). There was no evidence of mitotic figures, necrosis, or lymphovascular invasion. Immunohistochemical examination showed that the tumor cells were diffusely positive for chromogranin A and synaptophysin (Table 1). The Ki-67 LI was 0.5%. Accordingly, the tumor was diagnosed as a pancreatic NET G1.

Table 1 also shows the results obtained from the immunohistochemical evaluation of hormonal products and hormone receptors. The tumor cells were diffusely positive for glucagon and PgR, but negative for PP.

#### Adrenocortical adenoma in the left adrenal gland

Macroscopic examination showed that the nodular lesion in the left adrenal gland was 15 × 12 mm in size, well-circumscribed, and with a yellowish-tan coloring. Microscopically, the tumor was composed of both spongiocytic cells with a clear lipid-rich cytoplasm and lipid-poor compact cells with an eosinophilic cytoplasm (Fig. 3c, d). There was no evidence of mitosis, tumor necrosis, or vascular invasion. The pathological diagnosis was an adrenocortical adenoma. The surgical margins were negative for tumor cells.

#### Gene mutation analysis

DNA analysis of a peripheral blood sample showed no mutations in exons 2–10 of the *MEN1* gene, exons 1–3 of the *VHL* gene, and exon 16 of the *RET* gene.

#### Follow up

The patient’s postoperative course was uneventful. No signs of recurrence or metastasis were detected during a 1-year follow-up period.

## Discussion

Table 2 summarizes the clinical features of the four patients with pancreatic GP, including our patient, identified to date [9–11]. The average age of these four patients was 63 years, and three (75%) were women. Interestingly, the tumors in all four patients were located in the head of the pancreas, but our patient was the only patient without abdominal symptoms, such as abdominal pain and jaundice. Although duodenal GP is characterized by a favorable clinical course and a low incidence of lymph node metastasis (6.9% of 173 patients [13]), with none showing distant metastasis, three (75%) of the four patients with pancreatic GPs had metastases, including two with lymph node metastases and one with distant metastasis to the sternum. In addition pancreatic GPs were larger (mean diameter: 35 mm) than duodenal GPs (24.2 mm). These results are consistent with the greater difficulty detecting early stage pancreatic than duodenal tumors. Furthermore, pancreatic GPs may have a higher potential for malignancy than duodenal GPs.

**Table 2:** Summary of clinical features of pancreatic gangliocytic paraganglioma

Reference	Year	Age (years)	Sex	Size (mm)	Clinical Presentation	Metastasis	Operation	Outcome (months)
Tomic et al. [11]	1996	74	Female	40	Abdominal pain, Diarrhea, Steatorrhea, Nausea, Vomiting,	(+) lymph node	PD	NER 20
Henry et al. [10]	2003	50	Male	25	Cholestatic jaundice	(+) sternum	PD, followed by sternal resection	NER 18 from the second operation
Liu et al. [9]	2008	60	Female	53	Fever, Abdominal pain, Jaundice	NR	PD	NER 12
Present case	2016	68	Female	22	None	(+) lymph node	PPPD	NER 12

*PD* pancreaticoduodenectomy, *PPPD* pylorus-preserving pancreaticoduodenectomy, *NR* not reported, *NER* no evidence of recurrence

Pancreatic paraganglioma is important in the differential diagnosis of pancreatic GP, as the epithelioid cell component of GP is morphologically and immunohistochemically similar to paraganglioma [15, 16]. Moreover, most pancreatic paragangliomas are nonfunctional and located in the pancreatic head [17]. To distinguish GP from paraganglioma, we therefore immunohistochemically stained the tumor in our patient for catecholamine-synthesizing enzymes and pancytokeratin (Table 1). Most paragangliomas, including the non-functioning type, are positive for catecholamine-synthesizing enzymes [20] but negative for pancytokeratin [15]. To date, the details of catecholamine-synthesizing enzyme expression in GP have not been studied. However, the epithelioid cell component of GP in our patient was negative for catecholamine-synthesizing enzyme expression, suggesting that the functionality of this cellular component of GP may differ from paraganglioma.

Expression of PgR and PP may differentiate “duodenal” GP from “duodenal” NET G1 [16]. Assessment of 12 duodenal GPs showed that the epithelioid cell component in 11 (91.7%) were positive for PgR and PP. In contrast, duodenal NETs G1 were uniformly negative for PgR and PP expression. To date, no study has assessed the expression of PgR and PP in pancreatic GP. Interestingly, the immunohistochemical profile of PgR and PP expression in this patient were similar in the epithelioid cell component of the pancreatic GP and the NET, a result that may help in differentiating between pancreatic and duodenal GP. Further analysis is required to confirm the usefulness of PgR and PP expression in the diagnosis and differentiation of pancreatic GP.

Contrast-enhanced CT revealed differences in the contrast-enhanced patterns of GP in the pancreatic head and NET G1 in the pancreatic tail. FDG PET has been considered inadequate for the diagnosis of well-differentiated NETs due to their very slow growth [21]. The GP in this case, however, exhibited high ^18^F–FDG uptake, indicating that ^18^F–FDG PET could be a practical tool for detecting pancreatic GPs or to differentiate pancreatic GPs from NETs.

Several somatostatin analogs (SAs), including octreotide, lanreotide, and pasireotide, have been shown to have therapeutic benefits in patients with advanced pancreatic NETs [18]. The effectiveness of SA therapy for patients with GPs is entirely unknown, as none of these patients to date has been treated with SA. The present study, along with a previously reported case of GP in the ampulla of Vater, showed that the epithelioid cell component was immunohistochemically positive for SSTR2A [22], suggesting that SA therapy may have benefit GPs. Further study is warranted.

The co-occurrence of three distinct endocrine tumors in one patient suggests the possibility of a genetic predisposition in this patient. The genetic and biological backgrounds of this peculiar combination of three different endocrine neoplasms remain largely unknown. Although we found nothing indicating an inherited condition, the patient should be carefully monitored to allow for the early detection of other endocrine-related neoplastic lesions.

## Conclusion

This report describes the fourth reported case of pancreatic GP. Compared with duodenal GPs, pancreatic GPs were larger and had a higher incidence of metastasis, suggesting a greater potential for malignancy. Therefore, the primary organ of GP may be an important prognostic factor.

## Abbreviations

^18^F–FDG: Fluorine-18 fluorodeoxyglucose
CA 19–9: Carbohydrate antigen
CEA: Carcinoembryonic antigen
CT: Computed tomography
Dupan-2: Pancreatic cancer-associated antigen-2
GP: Gangliocytic paragangliomas
LI: Labeling index
MEN 1: Multiple endocrine neoplasia type 1
NET: Neuroendocrine tumor
NF 1: Neurofibromatosis type 1
NSE: Neuron-specific enolase
PCR: Polymerase chain reaction
PET: Positron emission tomography
PgR: Progesterone receptor
PP: Pancreatic polypeptide
Pro-GRP: Pro-gastrin-releasing peptide
SAs: Somatostatin analogues
Span-1: S-pancreas-1 antigen
SSTR: Somatostatin receptor
SUVmax: Maximal standardized uptake values
VHL: von Hippel-Lindau disease
